# Pulse Pressure as a Hemodynamic Parameter in Preeclampsia with Severe Features Accompanied by Fetal Growth Restriction

**DOI:** 10.3390/jcm13154318

**Published:** 2024-07-24

**Authors:** Rachael Sampson, Sidney Davis, Roger Wong, Nicholas Baranco, Robert K. Silverman

**Affiliations:** 1 Division of Maternal-Fetal Medicine, Department of Obstetrics & Gynecology, State University of New York Upstate Medical University, Syracuse, NY 13210, USA; 2 Department of Public Health and Preventive Medicine, Norton College of Medicine, State University of New York Upstate Medical University, Syracuse, NY 13210, USA; 3 Department of Geriatrics, State University of New York Upstate Medical University, Syracuse, NY 13210, USA

**Keywords:** preeclampsia with severe features, fetal growth restriction, hemodynamics, pulse pressure, maternal health, fetal health, obstetrics, gynecology

## Abstract

**Background**: Modern management of preeclampsia can be optimized by tailoring the targeted treatment of hypertension to an individual’s hemodynamic profile. Growing evidence suggests different phenotypes of preeclampsia, including those with a hyperdynamic profile and those complicated by uteroplacental insufficiency. Fetal growth restriction (FGR) is believed to be a result of uteroplacental insufficiency. There is a paucity of research examining the characteristics of patients with severe preeclampsia who do and who do not develop FGR. We aimed to elucidate which hemodynamic parameters differed between these two groups. **Methods**: All patients admitted to a single referral center with severe preeclampsia were identified. Patients were included if they had a live birth at 23 weeks of gestation or higher. Multiple gestations and pregnancies complicated by fetal congenital anomalies and/or HELLP syndrome were excluded. FGR was defined as a sonographic estimation of fetal weight (EFW) < 10th percentile or abdominal circumference (AC) < 10th percentile. **Results**: There were 76% significantly lower odds of overall pulse pressure upon admission for those with severe preeclampsia comorbid with FGR (aOR = 0.24, 95% CI = 0.07–0.83). Advanced gestational age on admission was associated with lower odds of severely abnormal labs and severely elevated diastolic blood pressure in preeclampsia also complicated by FGR. **Conclusions**: Subtypes of preeclampsia with and without FGR may be hemodynamically evaluated by assessing pulse pressure on admission.

## 1. Introduction

Multiple studies have postulated that distinct subtypes of preeclampsia may exist [1,2,3]. The underlying pathology may vary, which likely includes maternal cardiovascular and placental pathologies that lead to a common end pathway presenting with hypertension and endothelial inflammation [4,5]. Despite decades of research, much remains unknown about the interplay between preeclampsia and fetal growth restriction (FGR), and the presence of growth restriction may point to the underlying pathology. While fetal growth restriction is more common in pregnancies complicated by preeclampsia, the majority of patients presenting with preeclampsia have normal fetal growth [6]. Growth restriction was previously included in the criteria for severe preeclampsia (presently referred to as preeclampsia with severe features and shortened herein as PECS), but this was removed in 2013 because the American College of Obstetricians and Gynecologists (ACOG) noted that suspected fetal growth restriction is not indicative of PECS and suggested that abnormal growth be managed similarly in women with or without preeclampsia [7]. Many clinicians and researchers have remained suspicious that the presence of fetal growth restriction can have implications for the prognosis and management of some patients, even though it is not useful as a general diagnostic criterion.

Most studies and all current guidelines have focused on the absolute systolic and diastolic blood pressures and their use as a diagnostic criterion for severe disease and as an indication for emergent treatment to prevent maternal morbidity and mortality [8]. Several recent studies have focused on the maternal hemodynamic response to individual antihypertensive agents [9,10,11,12,13]. Specifically, hypertensive antenatal patients with hyperdynamic circulation, evidenced by a higher cardiac index, higher heart rate, and lower vascular resistance, were shown to respond well to labetalol [14]. This furthers the evidence supporting the hypothesis that women with a hyperdynamic circulation benefit more from beta-blockade, whereas women with a vasoconstricted profile benefit more from vasodilation with nifedipine or hydralazine [15].

The goal of our study was to examine a cohort of patients with PECS as compared to the cohort of patients with PECS who also had FGR to assess whether basic hemodynamic parameters differed between the two groups.

## 2. Materials and Methods

### 2.1. Data Source

This was a retrospective cohort study of consecutive patients admitted to the Regional Perinatal Center of Central New York diagnosed with PECS identified using facility ICD-10 codes from January 2022 through March 2023. This study cohort is well after 2013’s publication of ACOG’s Executive Summary on Hypertension in Pregnancy, which updated the diagnostic criteria for preeclampsia to not consider the presence of fetal growth restriction. It reflects data well into the post-COVID-19 era. The hospital’s medical record was fully electronic in this time frame. Patients were included if they had a live birth at 23 weeks of gestation or higher. Multiple gestations and pregnancies complicated by fetal congenital and/or chromosomal anomalies were excluded. The syndrome of hemolysis, elevated liver enzymes, and low platelets (HELLP syndrome) was excluded. Diagnoses were confirmed by individual chart reviews by two physicians (RS and NB). Therefore, misclassification bias regarding the diagnosis of PECS was likely very low after individual chart review and validation. Data were abstracted from the electronic medical record by two team members (RS and SD) and included maternal demographics and medical history, gestational age at admission and delivery, characteristics of the hospitalization and delivery, maternal vital signs on admission to referral center, maximum systolic blood pressure during antepartum course at the referral center, maximum diastolic blood pressure during antepartum course at the referral center, systolic blood pressure immediately preceding delivery, diastolic blood pressure immediately preceding delivery, and laboratory values throughout admission. Maternal age, gestational age, and pre-pregnancy body mass index (BMI) were collected as continuous variables. Patient-reported race and ethnicity, insurance payor, highest educational degree, and the presence of pre-pregnancy diabetes and pre-pregnancy hypertension were reported as categorical variables. Data from the New York State perinatal data system electronic birth certificate were matched with the abstracted chart data to include self-reported demographic and socioeconomic factors and to validate maternal medical history and pregnancy complications.

### 2.2. Preeclampsia with Severe Features

PECS was defined by blood pressure measurements reaching or exceeding systolic levels of 160 mmHg or diastolic levels of 110 mmHg, with or without proteinuria (diagnosed via 24 h protein excretion > 300 mg in 24 h, or if the ratio of measured protein to creatinine in a single-voided urine measurement exceeded 0.3), thrombocytopenia (platelet count less than 100,000 per µL), impaired liver function (elevated blood levels of liver transaminases to twice the upper limit of normal for hospital laboratory), new development of renal insufficiency (elevated serum creatinine more than 1.1 mg/dL), pulmonary edema, or new-onset cerebral or visual disturbances. A dichotomized severe laboratory composite was created if any one of these lab criteria was present.

All patients admitted to the antepartum service with a diagnosis of PECS had an ultrasound estimation of fetal weight performed. FGR was defined as a sonographic estimation of fetal weight (EFW) < 10th percentile or abdominal circumference (AC) < 10th percentile.

### 2.3. Hemodynamic Parameters

#### 2.3.1. Blood Pressure

The first systolic blood pressure (SBP) and diastolic blood pressure (DBP) on admission were recorded, as were the maximum SBP and DBP during hospitalization and immediately prior to delivery. Admission SBP was dichotomized to >160 mmHg and admission DBP to >110 mmHg. Mean arterial blood pressure (MAP) on admission was calculated as MAP = DP + 1/3(SP − DP).

#### 2.3.2. Pulse Pressure

Pulse pressure (PP) was calculated as SBP − DBP. In the context of obstetrics, higher pre-pregnancy and first-trimester pulse pressure have been associated with the development of preeclampsia [16,17]. However, the precise definition of widened pulse pressure varies between studies [18,19,20], and meaningful cutoffs of what constitutes a “wide” versus “narrow” pulse pressure in pregnancy, particularly in those with hypertension, are not currently known. Previous studies have defined these categories based on the distribution of measurements or means within their respective cohorts [11,16,17,21,22]. In interrogating our cohort’s data, we chose to use the dichotomous cutoff of PP > 55 mmHg.

### 2.4. Data Analysis

Hemodynamic parameter outcomes were compared in patients with and without growth restriction. Bivariate tests were conducted with independent sample *t*-tests for continuous variables and chi-squared tests (or Fisher’s Exact for expected frequencies less than five) for dichotomous variables. Multiple logistic regression was performed for dichotomous outcomes of severe systolic blood pressure on admission (>160 mmHg), severe diastolic blood pressure on admission (>110 mmHg), severe range blood pressure at any time during hospitalization, severe range diastolic blood pressure at any time during hospitalization, severe laboratory abnormality on admission or at any time during hospitalization, and admission pulse pressure >55 mmHg. Covariates were selected to include previously identified risk factors for growth restriction and preeclampsia. These included maternal age, insurance payor, maternal race and ethnicity, level of education, parity, BMI, gestational age on admission, and pre-pregnancy diabetes and/or hypertension. All analyses were conducted using SPSS package 29th ed. (IBM/SPSS, Chicago, IL, USA) with two-tailed tests and a significance level of 0.05.

## 3. Results

**Figure 1:**
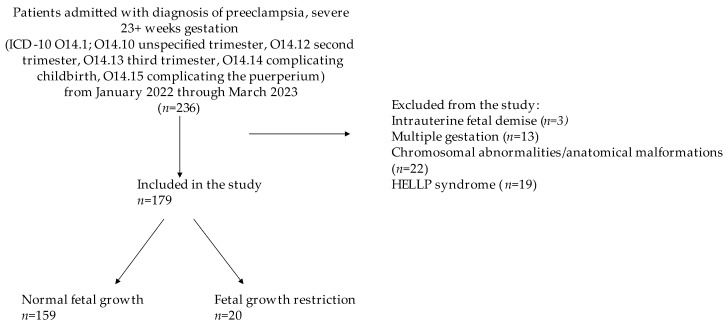
Flow chart for inclusion and exclusion criteria of study population.

A total of 236 patients at 23 weeks of gestation and above were diagnosed with PECS at our center in the time period specified. After excluding intrauterine fetal demise, multiple gestations, chromosomal abnormalities/genetic malformations, and HELLP syndrome, 179 patients with PECS were identified for inclusion in this study, 20 of whom had concomitant FGR (Figure 1). The baseline characteristics of the cohort are presented in Table 1. Race and ethnicity differed significantly between the two groups. The FGR group had a lower gestational age on admission, a lower BMI, and was more likely to be non-white or non-Hispanic. Parity, tobacco use, gestational age at preeclampsia onset, latency to delivery, mode of delivery, insurance, education level, and medical comorbidities did not differ between the two groups.

Table 2 highlights the results of the bivariate analysis via *t*-tests for continuous blood pressure variables. For the FGR group, the mean difference in pulse pressure on admission was lower by 8.13 mmHg (95% CI = 1.96–14.31, *p* = 0.010). The same did not hold true for mean arterial pressure; a bivariate analysis via *t*-test showed no significant relationship between mean arterial pressure (MAP) on admission for those pregnancies complicated by growth restriction and those with normal growth (mean difference 1.37 mmHg, 95% CI = −4.74–7.48, *p* = 0.658). There was no significant association of FGR with blood pressure or pulse pressure when examined at the time of maximum systolic and maximum diastolic pressures, nor at the time point immediately preceding delivery. Similarly, there was no significant relationship in the presence of severe laboratory abnormalities between the groups.

The results from the multiple logistic regressions are presented in Table 3. The adjusted odds of having an elevated systolic blood pressure (SBP) or diastolic blood pressure (DBP) on admission or at any time during the hospitalization preceding the delivery were not significantly associated with the presence of PECS with normal growth versus PECS with FGR. The odds of having a severe laboratory abnormality on admission or at any time during the hospitalization were also not significantly associated with growth restriction status. However, there were 76% significantly lower odds of >55 mmHg pulse pressure on admission for those with FGR (aOR = 0.24, 95% CI = 0.07–0.83).

In the same logistic regression, while FGR was not associated with severe lab abnormalities compared to the group with normal growth, each additional week of gestational age was associated with a 23% significant reduction in the odds of having a severe laboratory abnormality (aOR = 0.77, 95% CI = 0.67–0.89). Similarly, the presence of at least one instance of severely elevated diastolic blood pressure on admission was not associated with FGR but was associated with gestational age; each additional week of gestational age was associated with reduced odds of having severe diastolic hypertension by 13% (aOR = 0.87, 95% CI = 0.78–0.98).

## 4. Discussion

### 4.1. Pulse Pressure

We found that pulse pressure on admission was lower for patients with severe preeclampsia when the fetus was growth-restricted. This association was independent of maternal age, gestational age, BMI, and other potential confounding factors.

Pulse pressure is a surrogate measure of arterial compliance [23]. Wide pulse pressure is a sign of deteriorating cardiovascular health and carries an increased risk for mortality, disease progression, and adverse clinical outcomes in chronic diseases [24]. As pulse pressure rises above a normal level of 40 mmHg, the risk of cardiovascular conditions also rises; pulse pressures of 50 mmHg or more can increase the risk of heart disease and cerebrovascular accidents [25]. Higher pulse pressures are also thought to play a role in eye and kidney damage at the microvascular level [26]. It stands to reason that pulse pressure may affect the intricate vasculature of the placenta in a similar fashion. Pregnancy is associated with a reduction in uterine artery resistance, which facilitates trophoblastic invasion and the formation of a healthy, low-resistance placental circuit. When this fails, uterine artery resistance and total vascular resistance in the feto-placental circuit do not fall, and complications can develop, namely fetal growth restriction and/or preeclampsia [27].

Preeclampsia and fetal growth restriction are conventionally taught as pathologic disorders affecting the spiral arteries of the placenta. Historically, preeclampsia was thought of as “toxemia of pregnancy”, subscribing to the notion that abnormal perfusion of the placenta at the level of the spiral arteries led to the release of materials from the placenta [4]. This two-stage hypothesis began with a placental stage, which generated signals to the mother to cause a range of responses, resulting in the second stage, which culminated in the syndrome of preeclampsia [2]. A refined and extended model proposed syncytiotrophoblast stress as an important placental change, taking into account not only perfusion but also abnormal implantation, failed vascular modeling, and the effect of maternal immunologic factors such as antiphospholipid antibodies, thereby expanding potential pathways where fetal growth could be impaired [28]. Conceptually, a two-stage hypothesis of preeclampsia lends itself well to a classification of two subtypes of preeclampsia, namely, early-onset (less than 32–34 weeks gestation) and late-onset (greater than 32–34 weeks gestation), because preeclampsia at term is less often associated with FGR [1,29]. One-third of cases of preeclampsia are associated with fetal growth restriction [30], and these tend to be present with early-onset disease, for which delivery is indicated by 34 weeks when severe features are present [8]. However, even in an early setting, not all women with preeclampsia have growth-restricted infants. This begs the question that formed the impetus of our current investigational study: what can account for the fact that not all cases of PECS coexist with FGR?

Nearly twenty years ago, studies showed that pregnant women who later developed preeclampsia had elevated pulse pressure [17,31]. More recently, researchers have applied different methods for the detection of hemodynamic parameters in pregnancy [13,32,33]. These include the use of maternal echocardiography and the non-invasive Doppler method, as well as impedance cardiography and thermodilution. These studies provide research clinicians with valuable real-time insight into the equilibrium between a gravida’s stroke volume, heart rate, cardiac output, total vascular resistance, and blood pressure. However, our current standard-of-care national and international guidelines solely consider systolic and diastolic blood pressure levels without any consideration of the interplay of these absolute values on cardiac output or, perhaps more importantly, total vascular resistance. Understanding the differences in hemodynamic states may provide clues about the heterogeneous nature of hypertensive disorders during pregnancy [34].

The role of pulse pressure (PP) in characterizing the underlying pathologic state or predicting response to antihypertensive medication remains unclear. Previous work by Mullan et al. illustrated that pulse pressure did not predict response to antihypertensive medication; however, a wide pulse pressure predicted persistent, severe hypertension [11]. PP is proportional to the ratio of stroke volume (SV) to compliance (C) of the arterial tree [35]. PP widens in a high cardiac output state and can consequently serve as a readily available surrogate for SV without requiring an advanced ultrasound or Doppler hemodynamic monitor [36].

Emerging data and evolving concepts have led several authors to postulate that distinct preeclampsia phenotypes may exist beyond early-onset and late-onset syndromes and might be better characterized by maternal hemodynamic patterns [37,38,39]. These include a so-called vasoconstrictive state driven by low cardiac output coupled with high vascular resistance and another involving a hyperdynamic, high cardiac output intravascular volume-overloaded state [40,41]. There is likely overlap, with early-onset disease being more associated with the vasoconstrictive state compared to late-onset preeclampsia, which is more hyperdynamic in nature [34]. Additionally, there are data to support the distinction that, when adjusting for gestational age, maternal cardiovascular hemodynamic profiles differ between normally grown and growth-restricted fetuses [32,42,43,44,45]. We believe that our study findings support the hemodynamic hypothesis of distinct preeclampsia phenotypes.

### 4.2. Diastolic Hypertension and Severe Laboratory Parameters

We found that, while still widened greater than 55 mmHg, the admission pulse pressure for preeclampsia with severe features comorbid with a growth-restricted fetus was lower than the pulse pressure for those with a normally grown fetus. In our regression model, taking FGR into account with maximum diastolic blood pressure, advancing gestational age was found to lower the odds of diastolic blood pressure greater than 110 mmHg. This finding supports preeclampsia complicated by FGR as a hypodynamic state driven by diastolic hypertension, which in turn dampens pulse pressure against increased peripheral vascular resistance with a reduced cardiac output [12]. Similarly, when taking laboratory parameters into account in our regression, advanced gestational age lowered the odds of severely abnormal lab values for the FGR group. Therefore, PECS accompanied by FGR more closely resembles an early-onset and hypodynamic disease hallmarked by end-organ damage. We hypothesize that the factors driving the pulse pressure differential are related to central maternal hemodynamic parameters inherent to the growth-restricted group, suggesting, at least partly, a cardiovascular origin of the disease in addition to the long-held placental one.

### 4.3. Research Implications

Although we uncovered no significant association between FGR and pulse pressure parameters at other time points during hospitalization and preceding delivery, we hypothesize that this is likely secondary to the escalation of antihypertensive therapy per protocol. The algorithm for managing and controlling hypertensive emergencies in tertiary care centers is well delineated and adhered to. Such prompt treatment of acute-onset, severe hypertension decreases maternal morbidity and mortality [46,47,48]. Although the American College of Obstetricians and Gynecologists recommends timely recognition and treatment of severe hypertension to keep systolic blood pressure below 160 mmHg and diastolic blood pressure below 110 mmHg, there is no guidance regarding the tailored use of first-line medications [49]. This forms the basis of our proposed future research directions, whereby pulse pressure is proposed as a rapid and readily available indicator of maternal hemodynamics to guide antihypertensive choice, potentially resulting in a shorter time to resolution of and improved outcomes related to acute-onset, severe hypertension. Although numerous studies have compared various antihypertensive agents [50,51,52,53,54,55], randomization has not been stratified on the basis of suspected underlying maternal physiology, specifically in regard to maternal pulse pressure coupled with the presence or absence of FGR.

### 4.4. Strengths and Limitations

While central maternal hemodynamic monitoring techniques (Doppler non-invasive techniques, maternal echocardiogram, transthoracic bioimpedance) are not widely available at present on Labor and Delivery units, our study sought to elucidate hemodynamic parameters readily available to the practicing obstetric provider caring for patients presenting with preeclampsia with severe features in daily clinical practice. Although the absolute levels of blood pressure in our study did not suggest a difference between the subset with fetal growth restriction and normal growth, we did see a statistically significant difference when examining pulse pressure. This lends additional support to the emerging concept that the presence or absence of fetal growth restriction can pinpoint the specific underlying maternal cardiovascular dysfunction and therefore guide clinical management and pharmacotherapy.

A major strength of our study is the fact that pulse pressure is easily measured and readily available at all patient encounters, making it an attractive hemodynamic target for larger-scale clinical trials assessing pharmacologic intervention for hypertensive crises. Additionally, the center where our data were collected is an active clinical site, and the data reflect actual practice patterns and clinical management undertaken by medical staff practicing according to standardized diagnostic criteria. Our retrospective study has the potential for information or measurement bias, but the effect of this is felt to be lessened owing to our ability to cross-reference our electronic medical record with the state birth certificate data.

This study is limited by the small sample size resulting from our choice of study timeframe (14 months). Additionally, our tertiary care center accepts regional high-risk perinatal transports due to multiple gestations, fetal congenital anomalies, and HELLP syndrome; a total of 57 such patients were thereby excluded from the study sample. Given the retrospective study design, we are limited by the potential for unmeasured confounding and cannot prove causality. Future investigations of the effect of fetal growth restriction on hemodynamic parameters in patients with severe preeclampsia will need to be addressed in larger-scale studies, preferably in a prospective fashion coupled with maternal serum angiogenic markers.

## 5. Conclusions

Our findings support the hypothesis of different hemodynamics and origins for early preeclampsia. The presence or absence of fetal growth restriction may guide clinicians’ choice of antihypertensive agent based on individualized hemodynamic parameters and the complete clinical state of feto-placental health. Pulse pressure is a practical hemodynamic consideration given its ubiquity and ease of use on the clinical obstetric ward. Subtypes of preeclampsia with and without FGR may be hemodynamically evaluated by assessing pulse pressure on admission.

## Figures and Tables

**Table 1:** Baseline patient demographics and characteristics stratified by fetal growth.

Characteristic	Normal Growth (*n* = 159)	FGR (*n* = 20)	*p*-Value
Age (y)	29.7 ± 6.2	27.7 ± 7.4	0.185
Parity	0 (0–13)	0 (0–3)	0.676
BMI (kg/m²)	32.8 ± 9.3	26.8 ± 5.5	**0.008**
Tobacco use	18 (11.3%)	2 (10%)	1.000
Gestational age on admission (wk)	35.6 ± 3.6	33.9 ± 4.3	**0.027**
Preeclampsia onset < 32 wks	18 (11.3%)	5 (25%)	0.085
Latency to delivery	1 (0–22)	1 (0–25)	0.309
Cesarean delivery	97 (58.4%)	16 (80%)	0.088
Race and ethnicity			**<0.001**
White, non-Hispanic	111 (69.8%)	6 (30%)	
Black, non-Hispanic	20 (12.6%)	11 (55%)	
Hispanic	16 (10.1%)	1 (5%)	
Other	12 (7.5%)	2 (10%)	
Insurance			0.146
Private	69 (43.4%)	6 (30%)	
Medicaid	86 (54.1%)	12 (60%)	
Other	4 (2.5%)	2 (10%)	
Education level			0.209
Below high school	21 (13.2%)	1 (5%)	
High school or some college	64 (40.3%)	12 (60%)	
College and beyond	74 (46.5%)	7 (35%)	
Pre-pregnancy diabetes	15 (9.4%)	2 (10%)	0.936
Gestational diabetes	21 (13.2%)	1 (5%)	0.475
Chronic hypertension	9 (5.7%)	1 (5%)	0.931

FGR, fetal growth restriction. Data are in mean ± standard deviation, median (range), or *n* (%). Bold indicates statistically significant (*p* < 0.05).

**Table 2:** Bivariate analysis of continuous blood pressure variables.

Variable	Normal Growth (*n* = 159)	FGR (*n* = 20)	MD (95% CI)	*p*-Value
Admission systolic BP	151.99 ± 17.92	145.20 ± 19.06	6.79 (−1.66, 15.25)	0.114
Admission diastolic BP	90.71 ± 12.38	92.05 ± 11.27	−1.34 (−7.08, 4.40)	0.646
Admission MAP	111.14 ± 13.07	109.77 ± 12.87	1.37 (−4.74, 7.48)	0.658
Admission Pulse Pressure	61.28 ± 13.17	53.15 ± 13.42	8.13 (1.96, 14.31)	**0.010**

BP, blood pressure; MAP, mean arterial pressure; FGR, fetal growth restriction; MD, mean difference; CI, confidence interval. Data are in mean ± standard deviation. Units are in mmHg. Bold indicates statistically significant (*p* < 0.05).

**Table 3:** Adjusted odds of hemodynamic parameters by fetal growth.

Parameter	Normal Growth (*n* = 159)	FGR (*n* = 20)	aOR
Systolic blood pressure >160 mmHg on admission	55 (34.6%)	5 (25%)	0.71 (0.02, 2.49)
Diastolic blood pressure >110 mmHg on admission	11 (6.9%)	1 (5%)	0.66 (0.06, 7.76)
Severe systolic blood pressure during hospitalization	128 (80.5%)	15 (75%)	0.86 (0.21,3.49)
Severe diastolic blood pressure during hospitalization	40 (25.2%)	5 (25%)	0.42 (0.10, 1.66)
Severe lab abnormalities on admission	14 (8.8%)	0 (0%)	-
Severe lab abnormalities during hospitalization	20 (12.6%)	1 (5%)	0.18 (0.01, 2.64)
Admission pulse pressure > 55 mmHg	109 (68.6%)	7 (35%)	**0.24 (0.07, 0.83)**

FGR, fetal growth restriction; aOR, adjusted odds ratio. Data are *n* (%). Bold indicates statistically significant (*p* < 0.05).

## Data Availability

Dataset available on request from the authors.
